# Antimicrobial resistance – moving forward?

**DOI:** 10.1186/s12889-019-7173-7

**Published:** 2019-07-02

**Authors:** Marta Lomazzi, Michael Moore, April Johnson, Manica Balasegaram, Bettina Borisch

**Affiliations:** 1 Institute of Global Health, University of Geneva, 9 ch. des Mines, 1202 Geneva, Switzerland; 2 Food and Agriculture Organization of the United Nations, Viale delle Terme di Caracalla, 00153 Rome, Italy; 3 Global Antibiotic R&D Partnership (GARDP), 15 Chemin Louis-Dunant, 1202 Geneva, Switzerland; 4 World Federation of Public Health Associations (WFPHA), ch. des Mines, 1202 Geneva, Switzerland

**Keywords:** Antimicrobial resistance, Global charter, Intersectoral approach, Global public health

## Abstract

**Background:**

When microorganisms (such as bacteria or viruses) are highly exposed to antimicrobial drugs, they can develop the capacity to defeat the drugs designed to eradicate them. Long-term accumulation of adaptations to survive drug exposure can lead to the development of antimicrobial resistance (AMR). The success of antibiotics has led to their widespread overuse and misuse in humans, animals and plants.

**Main text:**

AMR is a global concern and solutions are not vertical actions in a single buy business model.

Even if a transectoral approach is key, there is a lack of multi-disciplinary partnerships that allow for strategic cooperation between different sectors such as the pharmaceutical industry, agro-alimentary complex, patient care and education, NGOs and research and development. Global public health voices should lead this integration to align the progress of existing AMR successes. Maintaining the public’s trust in preventive medicine, health systems and food production safety, together with public health driven, non-profit drug development, is a key factor. In its “Call for integrated action on AMR”, signed by about 70 national and international organizations the World Federation of Public Health Associations (WFPHA) called “on all governments, the private sector, non-governmental organizations, health professionals, public and private research organizations, and all stakeholders to ensure that public health remains at the centre of all policy and scientific endeavours in the field of antimicrobial resistance”.

**Conclusions:**

The “Global Charter for the Public’s Health”, developed by the WFPHA in association with WHO, is proposed in this article as a tool for implementation of complex public health actions such as AMR.

## Background

The development of antimicrobial drugs (e.g. antibiotics, antifungals, antivirals, antimalarials and anthelmintics) can alter the path of natural selection for microorganisms. Exposure to antimicrobials ensures that only random alterations in protein and genetic codes that allow microorganisms (e.g. bacteria, fungi, viruses, and parasites) to survive the drug are passed down in that organism’s genetic code [1]. Long-term accumulation of adaptations to survive drug exposure can lead to the development of antimicrobial resistance (AMR). The complacency induced by the success of antibiotics has led to their widespread overuse and misuse [2], accelerating the generation of multi-drug resistance [3].

AMR is a global concern as new resistance mechanisms are emerging and spreading globally, threatening our ability to treat common infectious diseases, resulting in prolonged illness, disability, and death [4]. In general, global public health challenges are not linear and solutions are not vertical actions in a single buy business model. Instead, multi-facetted, multi-stakeholder approaches are needed. This necessitates a great effort of coordination, political agreeableness, and mutual understanding. AMR is a complex, international concern that may endanger achievement of the Sustainable Development Goals [5].

The World Federation of Public Health Associations (WFPHA), together with the World Health Organization (WHO) and several international experts, developed a tool for implementation of complex public health actions titled the “Global Charter for the Public’s Health” (Charter) [6–8]. The Charter provides guidance for both ‘services’ (a group of core services - Protection, Prevention, and Promotion) and ‘functions’ (a group of enabler functions - Governance, Advocacy, Capacity, and Information). Both the basic services and the enabler functions are mandatory for the management of major worldwide health challenges such as AMR. The determinants of AMR and its outcomes are found on individual, regulatory, national, and international levels. Research, highlighting the danger of AMR is well established [4], yet the translation of this knowledge into sound political action has been rather slow. The Public Health community worldwide recognises and supports most of these initiatives; some multi-national and multi-stakeholder initiatives include ReAct, Joint Programming Initiative on Antimicrobial Resistance (JPI AMR), AMRindustryalliance, Global AMR R&D Hub, and several others. However, there is a lack of multi-disciplinary partnerships that allow for strategic cooperation between sectors such as the pharmaceutical industry, patient care and education, NGOs, and biomedical research and development. It is the task of public health to promote this integration to align the progress of existing AMR successes. More importantly, public health professionals must ensure that the social determinants that increase the likelihood of AMR are also put into focus.

**Fig. 1:**
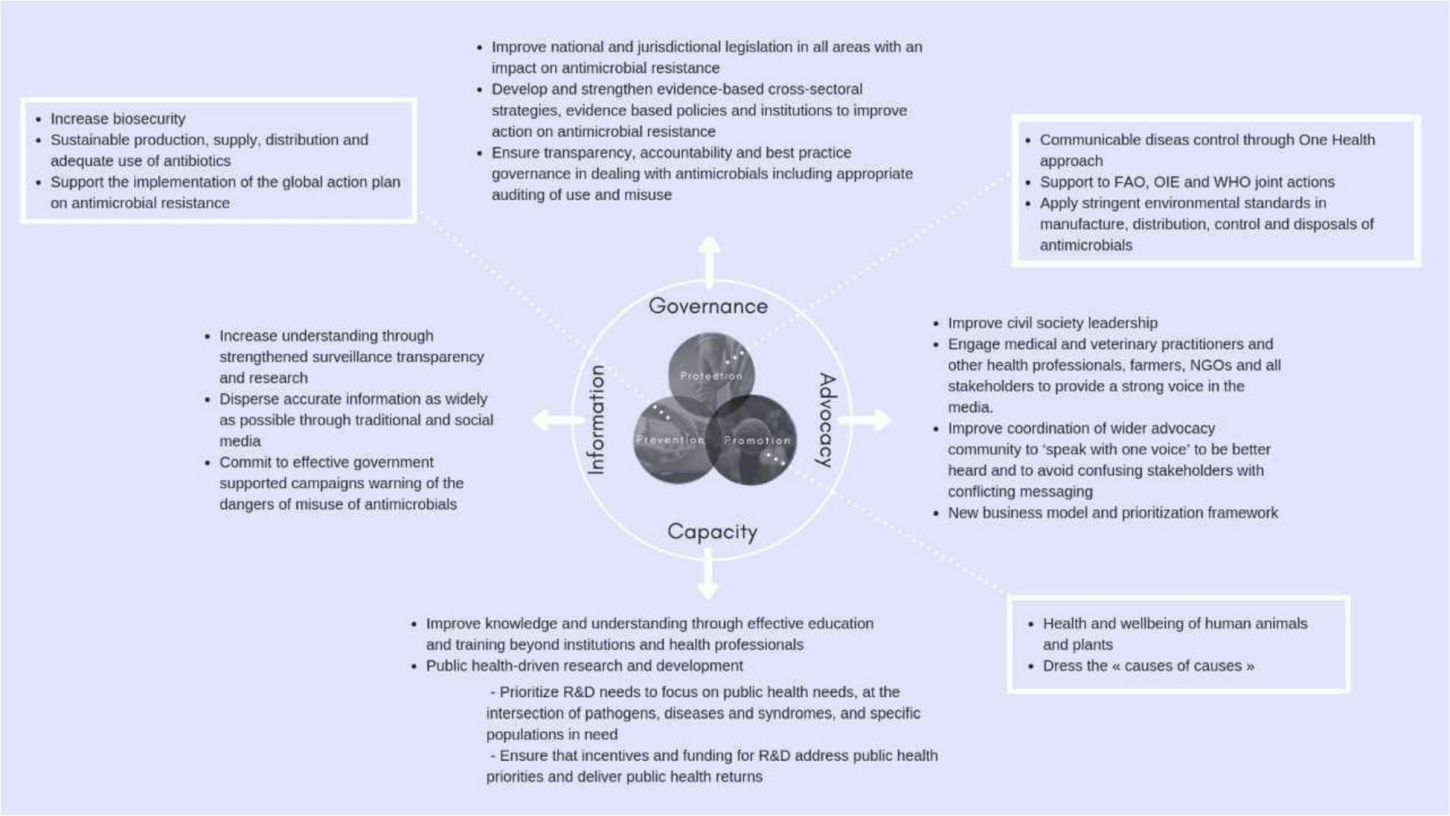
Title: Global charter for the Public’s Health applied to the AMR challenges. Legend: Key services and functions of the Global Charter for the Public Health that shall be implemented with a coordinated approach at global level to tackle AMR

This article highlights the need to break the glass ceiling closing the different gaps among human, animals and plants antimicrobials use and recommends innovative, coordinated, and comprehensive approaches to antimicrobial development and production. Each of the enabler functions in the Charter needs to be strengthened in a harmonised manner inclusive of more accountable data, to joint advocacy, capacity and awareness building up to effective global governance. Key examples of how Charter services and enabler functions can be implemented within this context are highlighted in the text and a broad overview is featured in Fig.1.

### AMR & human healh: how human health professionals can make the difference

A challenge for health practitioners, in addition to patient treatment, is the broader responsibility of preventing the very condition they are treating. Health professionals overwhelmingly support universal immunizations, recognizing the impact of vaccines in reducing the burden of disease of infections such as smallpox, measles, and polio. Antimicrobial drugs also play a key role in preventing infections such as pertussis, and are often administered before surgery to prevent infection following the operation; for this reason immunizations and antimicrobials have been fundamental in improving health across the socioeconomic spectrum. The important role that health professionals play in ensuring any government “takes proper account of recent evidence suggesting a wider responsibility for creating healthy societies” [9] has been understood for some time.

Prevention and treatment merge to meet the challenge of widespread, and worsening, resistance to anti-microbial drugs. Unless health care practitioners join with public health professionals as advocates there is a risk they will not be able to treat even some of the most common infections that are now so easily dealt with by a prescription of antibiotics. The WHO identified that “in 2016, 490 000 people developed multi-drug resistant TB globally, and drug resistance is starting to complicate the fight against HIV and malaria, as well”.(5) The Director of the Antimicrobial Resistance Secretariat at WHO, Dr. Marc Sprenger, identified “the importance of education, action, behavioural change and political commitment in combating AMR” [10]. Health professionals internationally must not bury their heads in the sand in the forlorn hope that the problem will fix itself.

The well-respected health and medical workforce is in a prime position to have considerable influence with governments locally, nationally and internationally. Developing the Charter enablers of capacity and advocacy would be key for the health workforce. They can learn to take action using published advocacy tools to becoming advocates. Such tools include building coalitions, selling the message, developing influential relationships, being opportunistic, generating short term wins and remaining persistent [11]. The adoption of “plain packaging of tobacco in Australia had the support of public health professionals, family doctors and the broad medical profession providing critical support for the government to maintain their courageous stance in the face of industry challenges in the High Court and in the World Trade Organization [12]”. Wernli and others have identified different ways that advocates frame AMR in order to influence governments including as “AMR as development”, “AMR as innovation”, “AMR as security” and more recently “AMR as one health”(9). This framing provides an insight into the way that public health advocates seek to influence government to provide sensible policies to deal with AMR. The global support to WFPHA “Call for integrated action on AMR” as well as the development of national and international Hubs such as Global AMR R&D Hub, established by the German Federal Government, or CGIAR, which applies a One Health approach in low- and middle-income countries to control agriculture-associated AMR risks, are a move in the right direction for gaining multi-sectorial engagement of professionals and improved coordinated advocacy that need to be done and strengthened [13] [14] (Fig. 1).

In providing this framing, Wernli et al. provide an illustration of why no health professional should be sitting on their hands in the hope anti-microbial resistance will solve itself. Framing the issue in different ways is more likely to reach a receptive ear of those in power. Therefore, when each and every one of us at least takes a small action to influence others, we contribute to a healthier future.

### AMR & animal and plants health: closing the gap with human health

As AMR affects human, animal, plant, and environmental health, a One Health approach is required to tackle its complexities. A 2017 country self-assessment survey report [15] undertaken by the Food and Agriculture Organization of the United Nations (FAO), World Organisation for Animal Health (OIE) and World Health Organization (WHO), found that more measures are being taken against AMR in the human health sector than in the animal health and agriculture sectors; including education, surveillance, monitoring, and regulation of Antimicrobial Use (AMU) and AMR. The environment and plant sectors are often unrepresented in national multi-sectoral AMR committees and there is substantially less data on these sectors [16].

Access to and appropriate use of effective antimicrobials are vital to produce safe and nutritious foods, as well as to ensure terrestrial and aquatic animal health and welfare. To retain efficacy of antimicrobials and limit environmental contamination, the overall use of antimicrobials should be reduced across all sectors while continuing to be affordable, accessible, and available in times of need. Minimization of AMU in animal production requires investment in and promotion of sustainable production practices including regular vaccine schedules, hygienic husbandry and biosecurity practices, and quality nutrition to decrease overall disease incidence. In addition, appropriate global surveillance mechanisms have to be put in place [17] as done by several single countries, but only the coordinate effort will be successful [18]. Antimicrobial use linked to growth promotion must be phased out, especially those that are critically important for human health. Research and innovation within the animal feed and animal health industry should also be promoted.

Antimicrobials are also used to treat bacterial and fungal diseases in plants. The quantities and extent of AMU in plant production is not well known, but antibiotics are approved for use for plant diseases in at least twenty countries [19]. There is also a paucity of information regarding the contribution of AMU to the emergence of AMR in plant microorganisms and the extent to which plant-origin resistant microorganisms colonize the human or animal gastrointestinal tracts and the degree this could represent a threat to human. While additional research in this area is necessary, reducing the need for and use of antimicrobials should reduce the risk of AMR in foods of plant origin [20]. Various measures can help reduce disease incidence and the need for antimicrobials such as using an Integrated Pest Management approach, improved biosecurity, and use of alternatives such as biological control and biorational products.

More resources and focus should be directed towards the food and agriculture sectors to better address AMR. These include raising awareness at all levels from farmers to policy makers, reducing the need for antimicrobials through improved husbandry and biosecurity practices, optimizing use of antimicrobials, strengthening regulatory frameworks, ensuring surveillance of AMU and AMR, access and availability of quality vaccines for animal use, and risk management at critical control points and monitoring progress to guide interventions and policies.

Increasing accountable information and develop more targeted communication as highlighted in the Charter would represent a milestone in increasing understanding, ownership and actions by communities up to governments to fight this treat (Fig. 1). Effective communication strategies shall consider all AMR aspects – humans, animals and plants, and focus on schematic images with scale and simplification to attract attention, story-telling to engage people to increase their understanding and modify belief, adopting straightforward wording without submerging the public’s with too many information [21] [22]. Embedding this effective communication strategy, the Charter may represent the guiding framework under the appropriate leadership to coordinate the awareness events and communication in partnership with the big coalitions and governments working on AMR from different sectors.

### R&D & public health driven, non-profit drug development

The alarming rate at which drug-resistant bacteria is increasing and outpacing the discovery of antibiotics calls for a different approach to prevent the estimated 700,000 deaths each year through antimicrobial resistance [23]. AMR is now a significant barrier to achieving Sustainable Development Goal 3 that seeks to ensure healthy lives and promote wellbeing for all. An additional concern is the current pipeline for new antibiotics and other therapeutic approaches which fails to address what the World Health Organization (WHO) has identified [24] as the biggest public health threats posed by increasingly drug-resistant Gram-negative bacteria [25], as well as tuberculosis. Furthermore, as new and remaining antibiotic developers struggle to mobilize financial resources, a coordinated effort between actors spanning research and development through to sustainable access is urgently needed to ensure both new and improved antibiotics remain available and effective to those who need them for generations to come.

An innovative approach proposed by the Global Antibiotic Research and Development Partnership (GARDP), is to focus on public health driven, non-profit antibiotic development [26] This means developing affordable new and improved treatments that address global public health needs, including indications not addressed by others due to perceived risks, challenges and cost. GARDP’s approach includes building sustainable access into its R&D strategies, ensuring access but not excess while reflecting the realities of clinical practice. It also takes into account the diversity of countries’ national health systems’ challenges and levels of economic development.

GARDP’s current Research and Development (R&D) efforts focus on developing and delivering antibiotics for Gram-negative drug-resistant infections in children, new-borns with sepsis, and sexually-transmitted infections (STIs) as public health priorities. The decision to focus on these is based on considering priority pathogens as identified by WHO, key populations’ health needs, and the unmet needs. This business model has the flexibility and capacity to enter at any point along the drug development pipeline, from early exploratory all the way through to patients. In addition to global public-health priorities, GARDP also bases its R&D strategies on clear target product profiles and scientific roadmaps.

Partnerships with a range of actors including industry, research institutions, academia, governments, civil society and not-for-profit organizations are key to GARDP’s non-for-profit approach. These guarantee resources can be effectively used and optimized, that the appropriate capacity and actors can be brought into accelerate product development, so as to significantly mitigate risk.

Antibiotic resistance can affect anyone of any age in any country [4]. Not-for-profit initiatives can work with a range of different actors to accelerate the development and sustainable use of new tools, helping to alleviate this threat. In order to do so, it is essential that adequate funding and resources are made available to ensure that a long-term and ambitious approach can be implemented for a robust pipeline to deliver sensible policies to combat AMR. This prioritisation of research, incentives and funding represent one of the key capacity aspects of the Charter to deliver public health returns (Fig.1).

### Conclusion

Antibiotics have revolutionized medicine as well as animal and plant production. However, overuse and misuse of antimicrobials, along with gaps in infection prevention and control, lack of quality standard medicines, and inadequate investments, have jeopardized breakthroughs in infectious disease treatment.

Dealing with this challenge requires a global effort. As with many of the world’s challenges, national borders are irrelevant - as are traditional “silo” approaches. However, even if this concept is generally accepted, we still observe fragmentation among the concerned sectors with lack of good governance, coordination, and disbursement of funds. Combatting AMR requires coherent and transparent governance, a trade-off between various economic interests, the right economic incentives, reliable instruments for research and monitoring, effective stewardship, and appropriate trade mechanisms. Maintaining the public’s trust in preventive medicine, health systems and food production safety is a key factor.

Legislation is a key component in addressing use and misuse of antimicrobials but too often fails to provide an appropriate regulatory answer due to the cross-sectoriality of AMR. Important efforts have been done by regions such Europe and countries such UK or Ghana but much remains to be done nationally and globally [27] [28] [29] . The global public health voice should lead the current policy discussions in the field of AMR to guarantee the comprehensive global approach, to emphasize the need to address the reality of healthcare systems and public health professionals worldwide within science, research and development, surveillance and epidemiology, healthcare delivery, universal healthcare, sustainable animal and agricultural production and consumption – all of which are required to sustainably address antimicrobial resistance for generations to come (Fig. 1).

In its “Call for integrated action on AMR” [30], signed by about 70 national and international organizations from different sectors, the WFPHA called “on all governments, the private sector, Non-Governmental Organizations (NGOs), health professionals, public and private research organizations, and all stakeholders to ensure that public health remains at the centre of all policy and scientific endeavours in the field of antimicrobial resistance”. To facilitate these joint global efforts, the Global Charter for the Public’s Health can pave the path to coordinate the key initiatives that incorporate acting from the individual empowerment to good governance for an effective global action.

## Data Availability

Not applicable.

## Abbreviations

AMR: Antimicrobial resistance
AMU: Antimicrobial Use
Charter: Global Charter for the Public’s Health
FAO: Food and Agriculture Organization of the United Nations
GARDP: Global Antibiotic Research and Development Partnership
JPI: Joint Programming Initiative on Antimicrobial Resistance
NGOs: Non-Governmental Organizations
OIE: World Organisation for Animal Health
R&D: Research and Development
STIs: sexually-transmitted infections
WFPHA: World Federation of Public Health Associations
WHO: World Health Organization
